# Beyond the Embryo: Rethinking the Genetics of Recurrent Implantation Failure in the PGT-A Era

**DOI:** 10.3390/genes17101170

**Published:** 2026-09-23

**Authors:** Charalampos Voros, Fotios Chatzinikolaou, George Papadimas, Ioannis Papapanagiotou, Ali Can Gunes, Athanasios Karpouzos, Kyriakos Bananis, Charalampos Tsimpoukelis, Maria Anastasia Daskalaki, Ioannis Pikrides, Nikolaos Thomakos, Panagiotis Antsaklis, Dimitrios Loutradis, Georgios Daskalakis

**Affiliations:** 1 Department of Obstetrics and Gynecology, ‘Alexandra’ General Hospital, National and Kapodistrian University of Athens, 80 Vasilissis Sofias Avenue, 11528 Athens, Greecepikrides114@gmail.com (I.P.);; 2 Laboratory of Forensic Medicine and Toxicology, School of Medicine, Aristotle University of Thessaloniki, 54124 Thessaloniki, Greece; 3 Athens Medical School, National and Kapodistrian University of Athens, 15772 Athens, Greeceloutradi@otenet.gr (D.L.); 4 Department of Obstetrics and Gynecology, Faculty of Medicine, Hacettepe University, Ankara 06100, Turkey; 5 King’s College Hospitals NHS Foundation Trust, London SE5 9RS, UK

**Keywords:** recurrent implantation failure, PGT-A, preimplantation genetic testing, karyotype, chromosomal microarray, thrombophilia, endometrial receptivity, IVF, infertility genetics

## Abstract

Background/Objectives: Recurrent implantation failure (RIF) affects many women undergoing IVF, but its genetic basis remains poorly defined. Most studies linking genetic or chromosomal factors to RIF were conducted before preimplantation genetic testing for aneuploidy (PGT-A) was widely used, when embryonic aneuploidy could not be reliably excluded. This review asks whether that evidence still holds now that PGT-A allows embryo aneuploidy to be excluded, at least analytically, as the default explanation for failed transfers. Methods: We reviewed the literature on parental karyotype abnormalities, chromosomal microarray findings, thrombophilia-related gene variants, and endometrial receptivity testing in women with RIF, comparing pre-PGT-A and post-PGT-A era studies where available. Results: Most foundational data on RIF genetics predate PGT-A and likely overstate the embryo’s contribution to implantation failure relative to maternal factors. Very few studies have re-evaluated RIF using strict, PGT-A-confirmed criteria for “true” implantation failure. Evidence for thrombophilia gene variants remains inconsistent across populations, and endometrial receptivity testing still lacks well-established genetic correlates. Conclusions: The genetic evidence base for RIF needs updating for the PGT-A era. A clear, PGT-A-confirmed picture of the genetic and chromosomal contributors to true implantation failure is still missing. Closing this gap should be a priority for future research in reproductive genetics.

## 1. Introduction

Recurrent implantation failure has no fixed definition. Clinicians have argued about it for thirty years. Every proposed cutoff (three transfers, four embryos, ten embryos) carries the same flaw. It ignores whether the embryo itself was ever the problem [1,2]. For most of that history, this flaw was unavoidable. Embryo ploidy could not be checked before transfer. A failed cycle might reflect a maternal problem. Or it might simply reflect an aneuploid embryo, silently discarded as “unexplained failure.” The two explanations looked identical from the outside. Preimplantation genetic testing for aneuploidy changed that. Once trophectoderm biopsy and chromosomal screening became routine, embryo ploidy stopped being a guess. A new clinical group emerged: women whose confirmed euploid embryos still failed to implant. This is a narrower, cleaner population. It is also a much smaller one than older studies assumed.

Ata and colleagues captured this shift well. They proposed defining RIF against the expected aneuploidy rate for a woman’s age, not against an arbitrary cycle count [3]. The logic is simple. Without knowing ploidy, the label “RIF” says almost nothing about biology. The 2022 Lugano consensus pushed the same point further. Its authors asked whether RIF, as classically defined, is a real clinical entity at all, or a statistical artifact created by transferring embryos of unknown chromosomal status [4]. Garneau and Young reached a similar conclusion from a different angle. They noted that without a shared definition, entirely different patient groups have been pooled under one name for decades [5].

The numbers make the point sharply. Gill and colleagues followed over 120,000 patients through five consecutive euploid blastocyst transfers [6]. Cumulative live birth rate reached 98.1%. Most patients labeled with RIF in the old literature, it turns out, simply had not yet transferred enough good embryos. Real, biologically distinct implantation failure (failure that persists despite confirmed euploidy) appears to be rare. This changes what a genetic explanation for RIF has to look like. If the embryo’s chromosomes are normal, meiotic non-disjunction cannot be the cause. Any genetic contribution left standing must work through something else, such as endometrial gene expression or maternal thrombophilic variants. Parental structural chromosomal rearrangements are a partial exception to this reasoning, addressed separately in Section 4: because a rearrangement’s embryonic consequence is a chromosomal imbalance, PGT-A is expected to flag the affected embryo as aneuploid directly, rather than leave it undetected within a confirmed-euploid cohort.

Almost none of the foundational literature on RIF genetics was built with this distinction in mind. Studies reporting elevated rates of parental karyotype abnormalities in RIF cohorts used definitions that could not rule out embryonic aneuploidy as the cause in any individual case. The thrombophilia literature shows the same limitation. Associations between polymorphisms such as PAI-1 4G/4G or Factor XIII Val34Leu and RIF vary by population, and none of the studies reporting them stratified by embryo ploidy. Endometrial receptivity testing is affected similarly: most validation studies enrolled mixed populations, in which some proportion of “failures” were plausibly unrecognized aneuploid transfers [7].

Our review asks a direct question. Which claims about the genetic architecture of RIF (parental chromosomal risk, thrombophilic variants, endometrial receptivity genes) were built on data that could not exclude embryonic aneuploidy? And which, if any, have been retested since PGT-A removed that confound? We treat these as separate questions, because they usually have separate answers. Where the post-PGT-A evidence is thin, we say so. Our goal is not another catalogue of candidate genes. It is an audit: how much of the genetic story we tell about RIF survives once the embryo is no longer a suspect. The genetic and molecular mechanisms examined in this review, together with their reported associations and the current availability of PGT-A-confirmed evidence, are summarized in Table 1.

## 2. Materials and Methods

This is a narrative review, not a systematic one, and that distinction shapes everything that follows. We did not register a protocol. We did not run a formal risk-of-bias assessment across every study. What we did instead was build an evidence audit around a single organizing question: did the study in front of us know, at the time of transfer, whether the embryo was chromosomally normal? That question turned out to split the literature more usefully than any thematic outline could.

We searched PubMed/MEDLINE, Scopus, and the Cochrane Library, combining the term “recurrent implantation failure” with each of the following in turn: PGT-A, preimplantation genetic testing, parental karyotype, chromosomal microarray, thrombophilia, endometrial receptivity array, HLA-G, and KIR. Searches were run between 15 January 2026 and 15 May 2026. Searches were not restricted by publication date on the lower end. Foundational papers from the 1990s and 2000s were kept wherever they introduced a concept, a polymorphism, or a definition still cited today, since removing them would have made the historical argument of this review incoherent. Because this review combined eight overlapping search-term pairs across three databases as part of an iterative, hypothesis-driven audit rather than a single systematic query, we did not track a formal count of records at each screening stage.

Each study that met our topic criteria was then read closely enough to answer one practical question: was embryo ploidy known and controlled for at the time of transfer, or was it not? Studies in the first group, the ones that could not exclude embryonic aneuploidy as an explanation for a failed cycle, we treat as pre-PGT-A evidence, regardless of their actual publication date. A study published in 2019 that never checked embryo chromosomes belongs here just as much as a study from 2003. Studies in the second group, where every transferred embryo carried a documented euploid result, we treat as PGT-A-confirmed evidence. This is the population in which a genetic claim about the mother, the endometrium, or the parental karyotype can actually be tested without the embryo muddying the result.

We applied this split separately to each of the four bodies of literature covered in this review: parental karyotype and structural chromosomal findings, thrombophilic gene variants, immunogenetic associations such as HLA-G and KIR, and the transcriptomic literature on endometrial receptivity. For each of these four areas, we asked the same pair of questions. First, what did the pre-PGT-A literature conclude, and how strong was that evidence taken on its own terms? Second, has any PGT-A-confirmed study since retested the same association? In several cases, no such study yet exists for the second question, and we state this explicitly rather than filling the gap with inference.

On inclusion, we kept human studies enrolling IVF or ICSI populations with some stated definition of RIF, however loosely specified, since a great deal of this literature predates any consensus definition at all. We included original research articles, systematic reviews, and meta-analyses that described their own search methodology in enough detail to be evaluated. Narrative reviews were used only to provide background, historical framing, or mechanistic explanation, never as standalone evidence for a prevalence figure or an association. Animal models and in vitro or cell-culture studies appear only where they help explain a molecular mechanism, such as the role of PAI-1 in trophoblast invasion or the transcriptomic basis of the window of implantation. They are never cited as evidence that a given mechanism operates in human RIF, since that would blur exactly the distinction this review is trying to preserve.

We excluded case reports as evidence for any claim about prevalence, frequency, or population-level association, since a single favorable outcome after an intervention cannot support a generalizable conclusion. We kept a small number of case reports where they illustrated a genetic mechanism with unusual clarity, most notably reports of successful pregnancy following PGT-SR in couples carrying a complex parental chromosomal rearrangement, since these cases demonstrate a mechanism that population-level statistics tend to obscure. We also excluded studies where the stated definition of RIF was so vague that it was impossible to judge whether embryo ploidy played any role in the reported failures, since including them would have defeated the purpose of the classification described above.

Finally, a limitation worth stating directly. Because embryo-ploidy status is not always reported explicitly, even in otherwise well-designed studies, our classification into pre-PGT-A and PGT-A-confirmed categories sometimes required inference from methods sections rather than an explicit statement by the original authors. Where that inference was uncertain, we note the uncertainty in the relevant section rather than assigning the study to either category by default.

## 3. The Shifting Definition of RIF: From Embryo-Blind to Embryo-Aware Diagnosis

Implantation is not a single event. It is a negotiation between two independent biological systems that happen to arrive at the same place at the same time. One is the blastocyst, carrying its own developmental program and its own chromosomal complement, fixed at fertilization and unchangeable afterward [20]. The other is the endometrium, a tissue that transforms itself across a narrow window of days into a state competent to receive that blastocyst, then closes again. Successful implantation requires both systems to be functionally normal and temporally synchronized. Failure can originate on either side, or in the mismatch between them. For most of the history of reproductive medicine, clinicians had a reasonable way to assess the second system and almost no way to assess the first [21].

This asymmetry shaped everything that followed. Embryo quality, in clinical practice through the 1990s and 2000s, meant morphological grading: cell number, fragmentation, symmetry, the rate of cleavage, and later, blastocyst expansion and the appearance of the inner cell mass and trophectoderm [22]. These are useful predictors of developmental competence in a general sense. They are poor predictors of chromosomal normality. A blastocyst can reach expansion on schedule, grade as top quality by every visual criterion available, and still carry a full trisomy or a complex mosaic karyotype incompatible with ongoing development [23]. The disconnect between visible morphology and underlying ploidy is not a small caveat to the older literature. It is close to a structural flaw. Every study built on morphological selection alone was working with a population in which an unknown, often substantial, fraction of “good quality” transferred embryos were chromosomally abnormal from the start.

The molecular reason for this disconnect lies in when and how aneuploidy arises. Most aneuploidy in human embryos originates in the oocyte, during meiosis I or meiosis II, through errors in chromosome segregation that become more frequent with maternal age as the cohesion complexes holding sister chromatids together weaken over the decades an oocyte spends arrested in prophase I [24]. A smaller but clinically important fraction arises post-zygotically, during the first mitotic divisions of the early embryo, producing mosaic embryos in which different blastomeres carry different chromosomal complements. Neither process leaves a visible signature on gross morphology in most cases [25]. Morphological grading assesses the output of transcriptional and metabolic programs operating downstream of the genome; it says very little about the genome’s numerical integrity itself. This is precisely the gap that chromosomal screening was designed to close, and precisely the gap that the pre-molecular RIF literature could not close [26].

The early clinical definitions of RIF were built without any awareness of this gap, because the technology to close it did not yet exist at scale. Coughlan and colleagues defined RIF as the failure to achieve a clinical pregnancy after at least four good-quality embryos, transferred across a minimum of three fresh or frozen cycles, in a woman under the age of forty [1]. The ESHRE PGD Consortium’s 2005 best-practice guidelines used a structurally similar logic: more than three good-quality embryo transfers, or the transfer of at least ten embryos across multiple cycles, without achieving pregnancy [2]. Both definitions count embryos and grade them morphologically. Neither asks, because neither could ask, whether those embryos were euploid. A woman who transferred eight aneuploid but morphologically excellent embryos and a woman who transferred eight euploid embryos that failed for genuinely maternal reasons would, under either definition, receive the identical diagnosis of RIF. Yet these are two biologically distinct situations, one embryonic in origin and one not, and no retrospective statistical analysis can separate them if ploidy was never measured [27].

The introduction of trophectoderm biopsy combined with comprehensive chromosomal screening, first through array comparative genomic hybridization and then through next-generation sequencing, changed the resolution at which this question could be asked [28]. For the first time, it became possible to know, before transfer, whether a given blastocyst carried a euploid chromosomal complement across all twenty-four chromosome types. This is not a trivial technical upgrade. It is the difference between inferring embryo quality from a proxy variable and measuring the variable that actually matters. Once ploidy could be checked directly, researchers could finally separate two populations that older definitions had collapsed into one: women who kept encountering aneuploid embryos by chance or by age-related biology, and women whose confirmed euploid embryos failed to implant regardless. Only the second group offers any genuine window into maternal, endometrial, or non-embryonic genetic contributions to implantation failure. The first group’s failures are fully explained by the embryo, and no maternal factor needs to be invoked at all [29].

Ata and colleagues built this insight directly into a revised definition of RIF, replacing an arbitrary embryo count with a probabilistic model grounded in developmental biology [3]. Their approach calculated the expected aneuploidy rate for a woman’s age, using published data on how blastocyst euploidy rates decline with advancing maternal age due to the accumulating meiotic errors described above, and used this age-specific rate to model the number of euploid-equivalent embryo transfers required before a defined cumulative implantation probability, set at 95%, could reasonably be expected. The output was striking. No single age category reached that threshold with fewer than six or seven transferred embryos. Under the older embryo-counting definitions, a substantial number of women labeled with RIF after three or four failed cycles had, in strict probabilistic terms, simply not yet been given a fair statistical chance. Their diagnosis reflected an artifact of counting, not a demonstrated maternal pathology.

The 2022 Lugano consensus extended this argument to its logical conclusion, moving from a call for better definitions to a direct challenge of whether RIF constitutes a distinct biological entity at all [4]. The consensus authors reviewed the post-PGT-A literature systematically and concluded that much of what had been labeled recurrent implantation failure was more accurately described as a statistical mirage: an expected consequence of transferring a limited number of embryos against a background aneuploidy rate, rather than evidence of an underlying maternal or endometrial defect [4]. Garneau and Young reached a compatible conclusion from a different angle, arguing that in the absence of a shared, biologically grounded definition, fundamentally different patient populations, some with genuine embryonic causes of failure and some with genuine maternal causes, had been studied interchangeably under a single diagnostic label for decades [5]. This, they argued, is sufficient on its own to explain much of the inconsistency and non-replication that characterizes the RIF literature, independent of any question about which specific genetic or molecular factors are actually at play.

The most direct empirical test of this argument came from Gill and colleagues, in a study whose scale allowed the question to be answered with unusual clarity. The authors followed more than 120,000 patients through as many as five consecutive euploid blastocyst transfers, tracking cumulative live birth rate at each successive transfer, restricted throughout to embryos with confirmed euploidy by PGT-A [6]. Cumulative live birth rate reached 98.1% by the fifth euploid transfer. Framed mechanistically, this result means that when the embryo’s chromosomal status is genuinely known and controlled for, the ceiling on eventual reproductive success is very high, and a persistent, biologically fixed maternal or endometrial barrier to implantation, one that would remain regardless of how many euploid embryos are offered to it, appears to affect only a small minority of patients. Most implantation failure, once the embryo is removed as a variable, resolves itself given enough attempts, and a persistent maternal or endometrial barrier appears to affect a minority of patients rather than the majority once labeled with RIF under older, embryo-blind definitions. That minority is not negligible: even the residual proportion of patients, under 2% of the more than 120,000 followed by Gill and colleagues, who had not achieved a live birth by the fifth confirmed-euploid transfer represents a real population, and the genetic and molecular mechanisms examined in the remainder of this review are addressed specifically to that population, not to the majority whose failure resolves with additional euploid transfers.

The ESHRE Working Group’s 2023 good practice recommendations settled on a more clinically workable compromise between Ata’s statistically rigorous 95% threshold and the practical needs of a fertility clinic, in which patients and physicians cannot reasonably wait for such prolonged sequences of transfers before initiating an investigation [3,30]. The Working Group proposed a 60% cumulative implantation probability threshold as the trigger for clinical evaluation of RIF, explicitly acknowledging that this represents a pragmatic balance rather than a claim about where true biological failure begins. This distinction between a statistically justified definition and a clinically actionable one is itself informative: it signals that the field has accepted the embryo-aware framework in principle, even where practical constraints prevent its full statistical rigor from being applied at the bedside.

It is worth being explicit about how PGT-A is used in this review. Its role here is definitional and analytical: it is used to classify existing studies by whether embryo ploidy was confirmed at enrollment, not as an endorsement of PGT-A as a routine clinical intervention. The clinical utility of PGT-A remains debated even outside the specific context of RIF, and professional bodies including the ESHRE Working Group have stopped short of recommending its routine use across all IVF populations [30]. Within the RIF population specifically, the most recent systematic review and meta-analysis on this question found that PGT-A-guided euploid transfer was associated with a significantly higher live birth rate per transfer and cumulative live birth rate than standard care (odds ratio 4.23, 95% CI 2.14–8.38, across four pooled studies), a result broadly consistent with the argument developed in this section that RIF may be substantially embryological in origin. The same authors caution, however, that further research is needed before PGT-A can be routinely recommended specifically for RIF management [31]. The analytic use of PGT-A-confirmed status throughout this review, as a filter for classifying the existing literature, should therefore not be read as an endorsement of PGT-A’s clinical benefit, which remains a separate and still-unsettled question.

This history is not background context to be read once and set aside. It functions as a filter for everything that follows in this review. Every study of parental karyotype, thrombophilic gene variants, immunogenetic polymorphisms, or endometrial receptivity gene expression discussed in the sections ahead was generated under one of these two paradigms, embryo-blind or embryo-aware, whether or not its authors framed it in those terms. A genetic or molecular association demonstrated in an embryo-blind cohort tells us, at best, that the association exists in a population containing an unknown mixture of embryonic and non-embryonic implantation failures. It cannot tell us whether that association holds in the narrower, biologically coherent population that the embryo-aware framework isolates. As the following sections show, in most areas of RIF genetics, that retest has simply not yet been done. Figure 1 places these successive redefinitions of RIF on a single timeline, relative to the point at which trophectoderm biopsy and NGS-based PGT-A became part of routine clinical practice.

## 4. Parental Chromosomal Contribution Revisited

Parental karyotype occupies a strange position in this review, different from every other topic covered here. Thrombophilia and endometrial receptivity are proposed as maternal factors that act independently of the embryo [32]. Parental structural chromosomal rearrangements do not work that way. A balanced translocation in one partner does not damage the endometrium or interfere with implantation signaling directly. It damages gametogenesis, upstream, by disrupting normal chromosome pairing and segregation during meiosis. The result is not a defective endometrium receiving a normal embryo. It is a defective embryo, produced by an otherwise normal reproductive tract.

### 4.1. The Molecular Mechanism: From Balanced Rearrangement to Unbalanced Gamete

Normal meiosis I depends on homologous chromosomes finding each other, synapsing through the synaptonemal complex, and forming at least one chiasma per bivalent to hold them together until anaphase I. This machinery assumes that each chromosome has exactly one normal homolog to pair with [33]. A reciprocal translocation breaks that assumption. Two non-homologous chromosomes exchange terminal segments, and the carrier’s cells now contain four chromosomes, two normal and two derivative, that share partial homology across broken segments rather than complete homology across their full length. During pachytene, these four chromosomes cannot form two simple bivalents. They are forced into a cross-shaped quadrivalent configuration, in which each chromosome pairs with two different partners along different stretches of its length [34].

The quadrivalent is unstable at the point where it must resolve into two products at anaphase I. Several segregation patterns are geometrically possible, and the specific pattern followed determines whether the resulting gametes are chromosomally balanced. Alternate segregation, in which the two normal chromosomes go to one pole and the two derivative chromosomes go to the other, restores balance. Every other pattern does not [34]. Adjacent-1 segregation separates homologous centromeres from each other rather than keeping them together, producing gametes that carry a partial trisomy for one segment and a partial monosomy for another. Adjacent-2 segregation is rarer and produces a different but equally unbalanced combination. The rare 3:1 pattern sends three chromosomes to one pole and one to the other, producing gametes with even larger imbalances. Segregation pattern is not fully random; the physical geometry of the quadrivalent, which depends on the size and position of the translocated segments, biases the outcome toward certain patterns over others. But no translocation carrier achieves 100% alternate segregation. A meaningful fraction of gametes, sometimes a majority, carry an unbalanced chromosome complement no matter how many cycles are attempted [35].

Robertsonian translocations, involving fusion of two acrocentric chromosomes at the centromere, follow a related but distinct mechanism. The derivative chromosome pairs with its two corresponding normal homologs to form a trivalent rather than a quadrivalent, and this trivalent resolves at anaphase I into one of three patterns: alternate segregation, in which the two normal chromosomes go to one pole and the derivative to the other, producing balanced or chromosomally normal gametes; adjacent segregation, in which the derivative and one normal chromosome go to the same pole, producing an unbalanced gamete; or the rare 3:0 pattern, in which all three chromosomes go to the same pole. The same underlying principle applies as for reciprocal translocations: only specific segregation outcomes restore chromosomal balance, and the others do not [36]. Pericentric and paracentric inversions carry a third distinct mechanism. Pairing across an inverted segment requires the chromosome to form a loop structure so that homologous sequences can still align. A crossover event occurring within that loop produces recombinant chromatids carrying duplications and deletions rather than a simple exchange, again generating unbalanced gametes downstream [37]. The specific molecular consequence differs across these three rearrangement types, but the endpoint converges: a structurally normal reproductive system, with intact hormonal signaling, unremarkable folliculogenesis, and functionally normal spermatogenesis or oogenesis, can nonetheless produce a chromosomally abnormal embryo at a rate determined not by age but by the topology of the parental rearrangement.

This has a direct and easily missed consequence for how PGT-A results should be interpreted downstream. A woman transferring embryo after embryo that comes back aneuploid on PGT-A is not automatically experiencing age-related meiotic decline, the mechanism usually invoked by default, particularly if she is young. She may instead be repeatedly generating the unbalanced segregation products of a structural rearrangement carried by herself or her partner. Reciprocal translocations typically generate segmental, rather than whole-chromosome, imbalances, and genome-wide PGT platforms are capable of resolving these as segmental gains or losses distinct from uniform whole-chromosome aneuploidy, as already demonstrated for array-based platforms applied to translocation carriers [38]. Robertsonian translocations behave differently in this respect: their unbalanced segregation products are whole-chromosome imbalances, since the material lost in the centromeric fusion is limited to non-essential satellite sequences, and are therefore reported as ordinary aneuploidy calls indistinguishable, on a single embryo’s report, from a trisomy or monosomy arising from oocyte meiotic non-disjunction. In either case, a pattern that no single PGT-A report can supply on its own the same chromosome or the same segmental imbalance recurring across multiple embryos from one couple is itself a clinical signal that should raise suspicion of an underlying parental rearrangement, distinct from the more randomly distributed pattern expected from age-related non-disjunction. PGT-A can therefore detect, and in the segmental case increasingly characterize, the consequence of the underlying rearrangement. Confirming the mechanism and its precise breakpoints still requires parental karyotyping.

### 4.2. PGT-SR as the Corrective Layer PGT-A Cannot Provide

Closing this diagnostic gap requires a parental karyotype in the first instance, and ideally a targeted molecular test built around the specific rearrangement once it is identified. Preimplantation genetic testing for structural rearrangements uses this same logic in reverse [38]. Once a parental breakpoint is mapped, either by conventional cytogenetics or by higher-resolution methods such as breakpoint-spanning FISH probes or SNP microarray analysis, embryo biopsy specimens can be screened specifically for the predicted unbalanced segregation products of that rearrangement, distinguishing embryos carrying the balanced form from those carrying an adjacent-1, adjacent-2, or 3:1 product. This is mechanistically distinct from generic whole-chromosome PGT-A. It is targeted to the known geometry of a specific quadrivalent, not a blind screen across all twenty-four chromosome types [34,38].

The case reported by Upadhyay and colleagues makes the clinical stakes of this distinction concrete rather than theoretical [39]. A couple with twenty years of infertility and ten failed IVF cycles had never undergone karyotyping despite the extraordinary duration of unexplained failure. Once high-resolution karyotyping identified a complex chromosome rearrangement in the male partner, involving three chromosomes rather than the simpler two-chromosome reciprocal translocation described above, and PGT-SR was designed around the specific breakpoints of that rearrangement, a viable pregnancy followed. Ten cycles of unexplained failure resolved once the correct molecular question, rather than a broader chromosomal screen, was finally asked.

This case also forces a qualification of the framework established in Section 3. For thrombophilia and endometrial receptivity, PGT-A confirmation genuinely isolates the population of interest, because those mechanisms operate independently of embryo chromosomal content. An embryo can be perfectly euploid and still fail to implant for a maternal reason unrelated to its own genome. Parental structural rearrangement does not behave this way. PGT-A does not remove the phenomenon from view when a rearrangement is present. It returns an aneuploid embryo result that, under an embryo-aware diagnostic framework, places the case in a different clinical category. A translocation carrier who transfers only unbalanced-derived, PGT-A-flagged aneuploid embryos accumulates a diagnosis of embryonic aneuploidy under the modern framework, rather than a diagnosis of RIF under the older embryo-blind one. The underlying genetic cause has not disappeared from the clinical picture. It has migrated into a diagnostic category that this review’s classification scheme would otherwise treat as fully explained by embryonic factors, and therefore as outside the scope of a review about non-embryonic contributors to implantation failure. That migration deserves to be stated explicitly, because it means the true prevalence of parental structural contribution to reproductive failure may be undercounted by both the old framework, which called it RIF without checking the embryo, and the new one, which correctly identifies the embryo as aneuploid but rarely traces that finding back to its parental origin.

### 4.3. Prevalence Estimates: Why the Numbers Disagree

The prevalence estimates reported across the existing literature vary enough to demand a mechanistic explanation rather than a simple average, and part of that variation reflects genuine differences in what each study counted as a chromosomal abnormality rather than a difference in the underlying biology. De Sutter and colleagues, working with a large series of 615 patients who had already failed at least three IVF/ICSI cycles, found chromosomal abnormalities in 2.1%, a figure combining balanced structural rearrangements, mainly reciprocal translocations with a single inversion, with numerical or mosaic sex chromosome findings and one marker chromosome [8]. Mozdarani and colleagues, in an Iranian cohort of 221 individuals, reported an overall abnormal karyotype rate of 9.5%, but this figure blends a recurrent spontaneous abortion subgroup, 13.92% abnormal, with a separately reported recurrent IVF/ICSI failure subgroup, 7.04% abnormal; the latter is the figure actually comparable to the RIF-specific estimates from the other three studies [9]. Asgari and colleagues, also working in an Iranian population but restricting inclusion to couples with RIF specifically rather than a mixed RSA/RIF population, found 3%, predominantly balanced reciprocal translocations, with one inversion, one Robertsonian translocation, and one benign satellite polymorphism that some laboratories would not classify as pathogenic at all [10]. Petrovic and colleagues, in a four-year retrospective study of 100 patients, reported 9%, comprising balanced translocations, including one Robertsonian translocation, and pericentric inversions, together with additional sex-chromosome and marker-chromosome findings [11]. None of these four figures measures quite the same thing: they differ in whether numerical and mosaic sex chromosome findings are counted alongside balanced structural rearrangements, in whether benign polymorphic variants are included, and, for Mozdarani specifically, in whether the denominator is restricted to a RIF-relevant population or blended with recurrent pregnancy loss.

Some of that spread traces to population-level differences in recombination and mutation background rather than to methodology. Consanguinity elevates the background prevalence of balanced structural rearrangements segregating within extended families, since a rearrangement introduced generations earlier persists and re-encounters itself more readily in populations with higher rates of related unions, independent of anything specific to the fertility clinic sampling that population. Sample composition explains a further portion of the spread [40]. Mozdarani’s cohort combined recurrent spontaneous abortion with IVF/ICSI failure rather than isolating RIF as an independent category, and RSA populations carry, on independent mechanistic grounds, a higher structural rearrangement burden than RIF populations [9]. This follows directly from the biology of unbalanced segregation products: an embryo carrying a partial trisomy or partial monosomy from adjacent-1 or adjacent-2 segregation is frequently capable of achieving implantation and even early placentation, since partial imbalances are, on average, less immediately lethal to peri-implantation development than the larger whole-chromosome imbalances typical of maternal-age-related non-disjunction, but then fails during the first trimester as the developmental consequences of the imbalance accumulate. This produces a pregnancy loss rather than a failure to implant at all. Blending an RSA population, enriched for exactly this mechanism, into an RIF denominator will inflate the apparent RIF-specific prevalence of structural rearrangements beyond what a strictly implantation-focused cohort would show.

Cytogenetic resolution adds a further, purely technical source of variance. All four studies relied on conventional G-banding or GTG-banding, a technique whose resolution is generally limited to rearrangements spanning several megabases [41]. Submicroscopic translocations, small cryptic insertions, and balanced rearrangements with breakpoints falling within gene-poor or repetitive regions routinely escape detection at this resolution and would only be identified by chromosomal microarray or genome-wide SNP array analysis, both of which offer sub-megabase resolution but were not used in any of the four studies discussed here. This means every prevalence figure reported in this section, De Sutter’s 2.1% included, should be read as a floor rather than a ceiling. The true rate of parentally transmitted structural contribution to embryonic aneuploidy in these populations is very likely higher than conventional karyotyping alone can reveal.

### 4.4. How Much of This Evidence Has Actually Been Retested

None of this variation, however sensible its explanation, answers the question this review actually needs answered. Every one of these four studies, without exception, was conducted under the embryo-blind definition of RIF discussed in Section 3. None restricted its RIF cohort to women whose failed transfers involved embryos with confirmed euploidy in the strict sense established by Ata and colleagues, or empirically validated at scale by Gill and colleagues. This is not a minor methodological footnote [3,6]. It means the field does not currently know whether parental structural chromosomal abnormality rates are elevated in the narrower, biologically coherent population the embryo-aware framework isolates, the population in which a genuinely non-embryonic explanation for implantation failure is actually being sought. Given the mechanism described in Section 4.1, it is entirely plausible that most or all of the karyotype abnormalities detected across these four cohorts belong to patients who, had PGT-A been applied prospectively, would have generated PGT-A results consistent with recurrent embryonic aneuploidy of parental origin, which, under an embryo-aware diagnostic framework, would place these cases outside RIF in the sense this review is trying to isolate. If that classification were applied consistently, the evidentiary basis for recommending routine parental karyotyping specifically within RIF workups, as opposed to within the broader workup of recurrent embryonic aneuploidy of any cause, would look considerably thinner than the raw prevalence figures suggest [34,35,36].

This is not an argument against karyotyping. The Upadhyay case demonstrates precisely why the test, and the targeted PGT-SR it enables, remains clinically indispensable, arguably more so now than in the embryo-blind era, since a couple whose embryos are consistently flagged aneuploid under generic PGT-A now has access to a specific, mechanistically grounded, and potentially resolvable explanation if a parental rearrangement is present, where indefinite further cycling with standard PGT-A alone would not resolve it [39]. But it is an argument for precision in how the existing prevalence literature is cited going forward. What that literature demonstrates, robustly, is that structural chromosomal rearrangements are one real and mechanistically well-characterized cause of recurrent embryonic aneuploidy. What it has not yet demonstrated, because no study currently in the literature was designed to ask this specific question, is whether parental karyotype abnormality contributes independently to implantation failure once embryonic aneuploidy has already been excluded by direct, embryo-level testing.

## 5. Thrombophilic and Prothrombotic Gene Variants

Thrombophilia occupies the opposite mechanistic position from parental karyotype within this review. Where a structural chromosomal rearrangement acts upstream of the embryo, corrupting gametogenesis before fertilization ever occurs, a maternal thrombophilic gene variant is proposed to act entirely downstream of the embryo, at the maternal-embryonic interface itself, independent of anything happening inside the blastocyst’s own genome. This is, in principle, exactly the kind of mechanism the embryo-aware framework established in Section 3 was designed to isolate. A euploid embryo can, in theory, fail to implant because the local hemostatic environment at the implantation site is hostile to trophoblast invasion, regardless of how chromosomally normal that embryo is. Whether the existing literature actually demonstrates this, rather than merely proposing it, is the question this section has to answer.

### 5.1. The Molecular Basis: Coagulation, Fibrinolysis, and Trophoblast Invasion

Successful implantation requires extravillous trophoblast invasion through the endometrial epithelium and remodeling of maternal spiral arteries, a process that depends on a regulated balance between extracellular matrix degradation and its restraint, centered on the plasminogen activator system [42,43]. Plasminogen activator inhibitor-1 (PAI-1) is the primary physiological inhibitor of this system [44]. A common promoter polymorphism, 4G/5G, controls PAI-1 expression: the 4G allele reduces binding of a transcriptional repressor present at the 5G sequence, so 4G homozygotes show higher constitutive PAI-1 transcription and circulating protein [45]. Excess PAI-1 activity is proposed to produce a hypofibrinolytic, anti-invasive local environment, impairing trophoblast invasion into the decidua and spiral arterioles [44,45].

Two further pathways are proposed to act through related but biochemically distinct mechanisms. Factor XIII, the transglutaminase that cross-links fibrin at the maternal-fetal interface, carries a common Val34Leu polymorphism that accelerates its activation and alters fibrin architecture, though whether the resulting network favors or impedes trophoblast invasion remains mechanistically ambiguous [46,47]. Annexin A5 (ANXA5) forms a protective shield over the trophoblast surface that limits local thrombin generation; its M2 promoter haplotype lowers ANXA5 expression, promoting thrombin generation and fibrin deposition through altered membrane protection rather than the coagulation cascade directly [48,49]. PAI-1, Factor XIII, and ANXA5 are frequently grouped under the single term thrombophilia, but they are mechanistically distinct pathways converging, at most, on a shared downstream phenotype of altered local hemostasis, a distinction that matters when interpreting composite thrombophilia panels that lump them together.

### 5.2. The Clinical Association Literature: Consistent Signal or Population-Specific Noise

Zeng and colleagues conducted a systematic review and meta-analysis of the PAI-1 4G/5G polymorphism in RIF, pooling eight case–control studies comprising 1273 women (697 with RIF, 576 controls). The homozygous 4G/4G genotype was significantly associated with RIF, with an odds ratio of 2.79 (95% CI 1.53–5.08) [12], a substantial effect size consistent with the biological mechanism described above.

The Factor XIII Val34Leu literature is less unified: studies examining this variant alongside broader thrombophilia panels in mixed recurrent-miscarriage and RIF populations have reported elevated variant frequencies in affected groups, but with less consistency than the PAI-1 finding, and isolated Factor XIII studies remain scarce [50]. The ANXA5 M2 haplotype adds a further layer of population dependence: Rogenhofer and colleagues, in 63 German couples with RIF, found significantly increased RIF risk among M2 carriers, with comparable risk from maternal and paternal carriage, an unusual finding since a paternal effect on trophoblast phosphatidylserine-binding capacity would require expression from paternally derived placental tissue [13]. Independent studies in Northern European populations have failed to replicate an association with related pregnancy complications.

This pattern, a fairly robust association for PAI-1, a less consistent one for Factor XIII, and a population-dependent one for ANXA5, likely reflects genuine cross-population variation in allele frequency and linkage disequilibrium rather than pure noise: a true effect of similar magnitude can appear stronger, weaker, or non-significant depending on a population’s baseline allele frequency and statistical power [51].

### 5.3. Why Even a Real Association Remains Clinically Contested

Even taking the PAI-1 association at face value, its clinical translation has been resisted by the field’s own consensus bodies. The ESHRE Working Group’s 2023 recommendations noted that the role of inherited and acquired thrombophilia in RIF, despite decades of clinical practice built around empirical anticoagulant treatment, is increasingly challenged as an evidentiary matter [30]. Part of this skepticism rests on a gap between genetic association and functional demonstration: an odds ratio of 2.79 establishes a statistical association with RIF as an outcome, but does not establish that circulating PAI-1 is actually elevated in a clinically meaningful way in women carrying that genotype, nor that any elevation observed is sufficient to impair trophoblast invasion in vivo, as opposed to in the isolated biochemical systems where this pathway has been most directly characterized [12,44,45]. The mechanism is well established biochemically; its sufficiency to explain a clinical phenotype from a common polymorphism of modest individual effect remains a separate, less settled question.

A further complication is that hypofibrinolysis, whether through PAI-1, Factor XIII, or ANXA5, is more mechanistically suited to explaining pregnancy loss after successful implantation than failure to implant in the first place [52]. Impaired trophoblast invasion and spiral artery remodeling are well documented contributors to later placental insufficiency, preeclampsia, and intrauterine growth restriction, all manifesting after implantation has already occurred [42]. Whether the same mechanism acts early enough to prevent the initial attachment and invasion events of implantation itself is a distinct question the existing literature does not address, since most studies define RIF as an outcome without verifying at which step failure occurred.

### 5.4. The Missing Ploidy Stratification

None of the studies discussed in this section, including the Zeng meta-analysis, restricted their cohorts to women with confirmed euploid embryo transfers in the sense established in Section 3 [12]. This omission is arguably more consequential here than for parental karyotype, precisely because thrombophilia is proposed to act independently of embryo genotype: an unstratified cohort should still detect a true independent association, diluted but not eliminated by embryonic-aneuploidy noise. But this assumption of independence has never been tested. Maternal PAI-1 genotype could instead interact with embryonic factors, for instance, if hypofibrinolysis increases the implantation window’s tolerance for a marginally competent embryo, which would strengthen rather than dilute the association in an unstratified cohort, an interaction the existing case–control literature cannot detect because it never measured embryo ploidy.

What the field currently has, then, is a molecularly coherent, replicated association for one thrombophilic variant, PAI-1 4G/4G, across eight studies with a reasonably narrow confidence interval, alongside two other proposed mechanisms, Factor XIII and ANXA5, with weaker or population-dependent support, none tested in a population where embryonic aneuploidy has been ruled out [12,13]. The clinical caution reflected in the 2023 ESHRE recommendations is, on these terms, a calibrated response to a literature that has demonstrated statistical association without yet demonstrating either the developmental stage at which the mechanism acts or its behavior in an embryo-confirmed population [30].

## 6. Immunogenetic Contributions to Implantation Failure

The embryo is immunologically non-self: half its genome comes from the father, and it expresses paternal antigens the maternal immune system has never previously encountered. Implantation therefore requires the maternal immune system to actively tolerate, rather than simply ignore, these paternal antigens at a tissue interface specialized for that function.

### 6.1. The Molecular Logic of HLA-G

Most nucleated cells display classical HLA class I molecules that flag normal self versus infected or foreign cells to cytotoxic T cells and NK cells. The trophoblast instead downregulates classical HLA-A and HLA-B, retains HLA-C, and expresses a non-classical molecule, HLA-G, that is a poor activator of cytotoxic T cells and has low allelic polymorphism, consistent with a regulatory rather than peptide-presentation role [53]. Both its membrane-bound and soluble isoforms, generated by alternative splicing of a single transcript, engage inhibitory receptors on maternal immune cells, principally LILRB1 and LILRB2 (also known as ILT2 and ILT4), which act on decidual stromal cells and the maternal vasculature respectively, suggesting the same HLA-G signal may support both local immune tolerance and vascular remodeling [53,54,55].

The functional polymorphisms sit mostly outside the coding sequence: a 14-base-pair insertion/deletion in the 3′ untranslated region alters mRNA stability, with the insertion allele destabilizing the transcript, and Hu and colleagues, in a meta-analysis pooling multiple case–control studies, found this variant associated with lower soluble HLA-G concentration in seminal and blood plasma of couples undergoing IVF [14]. A promoter polymorphism at position −725 works through the same logic of altered expression level.

The clinical associations split in an informative way. Hu and colleagues found the maternal HLA-G*010101 allele and the paternal HLA-G*010102 allele each independently associated with RIF risk [14]. The paternal association is notable: it cannot work through the maternal immune system reading the mother’s own genotype, and instead must act through the paternally inherited allele expressed in placental tissue, a genuinely embryonic contribution operating through an immunological rather than chromosomal-copy-number mechanism. Not every polymorphism showed the same signal, however: the maternal 14 bp ins/del genotype alone, the −725 promoter polymorphism alone, and circulating sHLA-G concentration alone were not significantly associated with RIF in the general population. The association became visible only in specific contexts, such as the 14 bp ins/ins genotype in populations of Caucasian ancestry, a subgroup effect not replicated in other ancestries, consistent with baseline sHLA-G expression depending on genetic background at other interacting loci [14].

### 6.2. KIR and the Uterine NK Cell Decision

The dominant immune cell at the implantation site is a specialized uterine NK cell, phenotypically distinct from circulating blood NK cells, that carries killer immunoglobulin-like receptors (KIRs) reading HLA-C on the trophoblast surface; depending on which KIR engages which HLA-C variant, the resulting signal is inhibitory or activating [56]. The KIR gene cluster forms two haplotypes: haplotype A carries almost exclusively inhibitory genes, while haplotype B carries a mixed set including several activating KIR genes absent from haplotype A, giving each woman an AA, AB, or BB genotype that sets the baseline balance of inhibitory to activating signaling before any pregnancy-specific signal arrives [57]. HLA-C itself divides into two functional groups, C1 and C2, distinguished by a single amino acid that determines which KIRs engage it, with C2 preferentially recognized by several activating KIR genes found only on haplotype B [58].

KIR2DL4 is a partial exception, present on nearly all uterine NK cells regardless of haplotype and capable of recognizing soluble HLA-G directly, linking the KIR and HLA-G pathways at the molecular level [55,59].

Maftei and colleagues tested KIR haplotype directly against reproductive outcome in patients with RPL and RIF undergoing single embryo transfer. Women carrying the KIR AA haplotype had a significantly higher chance of miscarriage, with an adjusted odds ratio of 4.15 (95% CI 1.39–6.50, *p* = 0.032), compared to those who conceived spontaneously, while women carrying the BB haplotype had a significantly lower miscarriage rate (6.9%) and a correspondingly higher live birth rate (87.5%) [15]. The direction of this effect fits the receptor biology described above: an AA genotype, dominated by inhibitory signaling with no activating genes to counterbalance it, may under-license the cytokine secretion and vascular remodeling activity uterine NK cells contribute to during early placentation.

### 6.3. Adding a Layer: Peptide Processing Upstream of the Receptor

The interaction becomes more specific once maternal KIR genotype is combined with the paternally derived HLA-C genotype of the embryo. Piekarska and colleagues tested this combined system in 491 couples undergoing IVF against 322 fertile controls, adding ERAP1 and ERAP2, the aminopeptidases that trim the peptide cargo loaded onto HLA-C and thereby influence how strongly a given KIR engages it, into the model [16]. A specific maternal ERAP1 genotype (CC at rs27044) combined with a paternal HLA-C1C1 or HLA-C1C2 genotype was protective against RIF, with odds ratios of 0.343 and 0.442, respectively [16]. This means the relevant genetic risk is distributed across at least three interacting loci, maternal KIR, embryonic HLA-C, and maternal ERAP, rather than concentrated in any one, which may explain part of the inconsistency across smaller single-gene association studies in this area.

### 6.4. What This Evidence Actually Supports, and What It Does Not

None of the immunogenetic associations described here were tested in a population restricted to confirmed euploid embryo transfers, which matters differently here than for thrombophilia. Because HLA-G and HLA-C genotypes are embryonic, not purely maternal, an aneuploid embryo does not simply add unrelated noise: it could carry an entirely normal HLA-C or HLA-G genotype and still fail to implant for chromosomal reasons unrelated to these mechanisms, diluting any true signal. The reverse interaction is also plausible and untested: a maternal KIR AA genotype might specifically affect patients who are also more likely to be offered marginal or mosaic embryos, confounding the two mechanisms in a direction existing case–control designs cannot resolve.

The KIR-HLA-C-ERAP literature, taken as a whole, offers a mechanistically richer and more internally consistent case than the thrombophilia literature in Section 5, since the direction of effect at each layer, AA harmful, BB protective, specific ERAP1-HLA-C combinations protective, replicates and extends the underlying receptor biology. But the same structural gap applies: no study asked whether this association persists once embryonic aneuploidy has been ruled out by direct testing, and until it does, the immunogenetic contribution to RIF remains mechanistically well supported but empirically incomplete.

## 7. Molecular Genetics of Endometrial Receptivity

A euploid embryo still needs somewhere to land. The endometrium is not a passive surface. It is a tissue that changes its own gene expression profile on a schedule, opens a narrow functional window, and closes it again days later. Get the timing wrong, and a perfectly normal embryo meets a wall instead of a receptive lining.

### 7.1. The Transcriptomic Signature of the Window of Implantation

The endometrium moves through a defined sequence of states across the menstrual cycle: proliferative, early-secretory, mid-secretory, late-secretory. The window of implantation sits within the mid-secretory phase, classically placed around days 19 to 24 of a 28-day cycle, though the actual timing varies between women by several days [60]. Older methods tried to capture this transition through tissue morphology alone, looking for structural markers like pinopode formation on the luminal epithelial surface, or changes in integrin expression patterns, particularly integrin αvβ3, thought to mark the receptive apical membrane [61]. These markers correlate with receptivity in a general sense but have never reached the reproducibility needed for individual patient decisions. Two women prepared identically by hormone replacement therapy can have their true receptive window sitting on different calendar days, and a standard fixed-day embryo transfer will miss the window in one of them without anyone knowing it happened.

The endometrial receptivity array approach treats this as a gene expression problem rather than a histological one. Instead of grading endometrial tissue by microscopic appearance, the array measures expression of a defined gene panel known to shift specifically around the window of implantation, originally described as around 236 to 238 genes depending on the platform version, later refined into smaller targeted panels [62]. A computational predictor trained on this expression pattern classifies a biopsy as pre-receptive, receptive, or post-receptive relative to the day it was taken, and tells the clinician how many hours earlier or later the transfer should be scheduled in a subsequent cycle to land inside that specific patient’s actual window.

Newer platforms extend this logic with smaller gene sets. Opuchlik and colleagues used a beREADY test targeting 67 biomarker genes for endometrial receptivity, applied to endometrial biopsies from 68 women with RIF, defined in their study as at least three unsuccessful transfers of good-quality blastocysts of at least 1BB grade, and 49 controls undergoing IVF for idiopathic infertility or male factor alone [19]. Rather than a binary receptive/non-receptive call, this platform classified patients into four categories: pre-receptive, early-receptive, receptive, and post-receptive. Sixteen women in the RIF cohort fell into the pre-receptive category and fifty-four into early-receptive, meaning the large majority of this RIF cohort was not sitting where a standard fixed-day protocol would have assumed them to be [19]. The study also identified age as one of the strongest factors influencing endometrial receptivity in this cohort, an association that fits what is separately known about age-related decline in stromal decidualization capacity, a process distinct from the ovarian aging mechanisms that drive oocyte aneuploidy discussed earlier in this review.

The reproducibility of a single ERA result is itself an open question, and one case worth noting directly. Ota and colleagues reported a woman with RIF and chronic endometritis who underwent three separate ERA tests, and the three tests returned discrepant classifications of her window of implantation rather than a stable, repeatable result [63]. This raises a question the field has not fully resolved: whether receptivity classification reflects a fixed patient-specific trait, stable across cycles the way a genetic polymorphism would be, or a state that shifts cycle to cycle depending on hormonal preparation, inflammatory status, or technical variability in the assay itself. A discrepant repeat result is a very different problem from a discrepant genetic polymorphism call, and it complicates how confidently any single receptivity finding should be treated as diagnostic.

### 7.2. The Genes Doing the Work: LIF and Its Transcriptional Control

Behind the array’s expression signature sit specific genes with defined, mechanistically characterized roles in the attachment process itself. Leukemia inhibitory factor, LIF, is one of the most consistently implicated [64]. LIF is secreted by endometrial glandular epithelium precisely around the window of implantation, signals through the LIF receptor and gp130 coreceptor complex on both the endometrial epithelium and the trophectoderm, and activates downstream JAK/STAT3 signaling that promotes the initial attachment reaction, the first physical contact between trophectoderm and luminal epithelium that precedes invasion [65]. This is not a redundant pathway with a backup system standing by. In mouse models, loss of LIF signaling produces a complete block of blastocyst implantation despite otherwise normal embryo development, which is why LIF has remained a focal point for human receptivity research even though direct manipulation of the pathway in patients has not translated into an approved therapy.

LIF expression is not autonomous. It sits downstream of a specific transcriptional control network. Huang and colleagues identified Krüppel-like factor 12, KLF12, as a direct negative regulator of LIF transcription in human endometrial glandular and stromal cells [17]. KLF12 is expressed in glandular epithelium and stromal cells of the secretory-phase endometrium, and the same research group had previously shown, in earlier work cited within this study, that KLF12 also negatively regulates endometrial decidualization by transcriptionally repressing a distinct set of target genes involved in that separate process [66]. This means KLF12 sits at a regulatory node controlling two separate receptivity-related programs, attachment through LIF and decidualization through other targets, rather than acting on a single isolated pathway. In endometrial biopsies from women with RIF, the study found the opposite pattern from what normal cycling predicts: KLF12 protein levels were excessively elevated, and LIF expression was correspondingly decreased, compared to fertile controls (Huang et al., 2018) [17]. Mechanistically, this is a transcriptional repression problem, not a structural gene defect. The LIF gene itself is presumably intact in these patients. Its promoter is simply being silenced by a repressor that has failed to step aside on schedule, at exactly the cycle days when LIF secretion needs to peak.

This kind of finding illustrates something the earlier sections of this review could not, because karyotype and thrombophilia variants are fixed germline polymorphisms, present from conception and unchanging across cycles. A transcriptional regulatory defect like excess KLF12 is, at least in principle, a modifiable cellular state rather than a fixed genetic sentence, since transcription factor levels respond to upstream hormonal and paracrine signaling, and upstream signaling can in theory be targeted pharmacologically or through cycle-specific hormonal adjustment in a way a structural chromosomal rearrangement cannot be corrected. Whether any current intervention actually normalizes KLF12 or LIF expression in a clinically meaningful way in RIF patients remains untested at the level of interventional trial data.

### 7.3. Clinical Evidence: With and Without PGT-A Alongside It

The clinical literature testing ERA-guided transfer in RIF patients divides cleanly along exactly the axis this review has followed throughout: studies that also controlled for embryo ploidy, and studies that did not. Amin and colleagues offer one of the few studies to combine both tests directly. They enrolled 291 RIF patients, split into Group I, which did not receive ERA testing, and Group II, the ERA study group, which was further subclassified by test result into receptive and nonreceptive subgroups, and additionally screened a subset of patients for embryo ploidy using PGT-A before transfer [18]. In the nonreceptive endometrial subgroup, meaning the patients whose initial ERA result would have led to a mistimed transfer under a standard fixed-day protocol, adjusting the transfer schedule to match the ERA-determined window produced a clinical pregnancy rate of 73.5% and an implantation rate of 78.6% [18].

The same study reported a separate comparison specifically in patients using donor oocytes. In that subgroup, clinical pregnancy rate was 64% in the receptive ERA group and 65% in the nonreceptive group after personalized timing, with implantation rates of 65% and 74%, respectively [18]. The near-equivalence of outcomes between receptive and nonreceptive groups once personalized timing was applied is itself informative: it suggests the intervention, not the baseline classification, is what levels the outcome, consistent with the idea that a correctly timed transfer can overcome an initially unfavorable receptivity classification. The authors’ own interpretation of the PGT-A component is worth stating plainly: combining PGT-A with ERA, in their framing, rules out the genetic contribution from the embryo side, leaving the receptivity intervention to address the maternal side specifically [18].

This is a meaningfully different study design from most of the ERA literature, which tests receptivity-guided transfer without any parallel embryo ploidy data at all. A positive result from an ERA-guided protocol in a cohort where embryo ploidy was never checked cannot distinguish two explanations that look identical from the outside: the transfer succeeded because the endometrium was now correctly timed to receive a normal embryo, or the transfer succeeded because, by chance, that particular cycle happened to use a euploid embryo regardless of timing, and the ERA intervention contributed nothing at all. Only a design like Amin’s, with ploidy data available even partially, begins to separate these two possibilities, and even there the PGT-A screening was applied only to a subset of the full cohort rather than universally, leaving part of the sample still subject to the same ambiguity.

Two further studies have since applied PGT-A alongside ERA testing at larger scale, though neither was designed prospectively. Cozzolino and colleagues stratified 2598 RIF patients by severity and by the testing received, PGT-A, ERA, PGT-A plus ERA, or neither, and found that PGT-A alone was associated with higher implantation and ongoing pregnancy rates in moderate RIF, while adding ERA testing did not improve outcomes in either severity group [67]. Neves and colleagues compared ERA-guided transfer against standard timing in a smaller cohort restricted to patients with PGT-A-confirmed euploid embryos, and likewise found no improvement in pregnancy outcomes from ERA testing, with a possible reduction in outcomes in the donor-oocyte subgroup [68]. Both studies therefore did apply PGT-A to at least part of their cohort, addressing the ploidy-confound concern this review has raised throughout, and both still found no clinical benefit from ERA-guided timing, a result that argues against, rather than for, the receptivity biomarker’s current clinical readiness.

### 7.4. Why Receptivity Remains the Least Resolved Genetic Component

Even granting the partial or retrospective ploidy control applied by Amin, Cozzolino, and Neves and their colleagues, no study reviewed in this section prospectively restricted its entire cohort to confirmed euploid transfers before evaluating a receptivity gene panel, KLF12 expression, or ERA classification against outcome [18,67,68]. This gap matters more here than in any of the three domains covered so far. Parental karyotype acts upstream of the embryo. Thrombophilia and immunogenetic factors act at the interface but through variants fixed at conception. Endometrial receptivity is different again: it is a maternal tissue state that changes cycle to cycle, and a mistimed transfer of a genuinely euploid embryo would look, on outcome data alone, indistinguishable from a well-timed transfer of an embryo that happened to be aneuploid.

The reproducibility problem illustrated by the Ota discrepancy case compounds this further [63]. A germline polymorphism, once genotyped, does not need to be retested each cycle. A receptivity classification, in this single reported case, apparently can shift across repeated tests in the same patient without any change in the underlying condition being treated. If receptivity classification is this unstable in some patients, then a single ERA result used to guide a single transfer decision carries a different kind of uncertainty than a KIR genotype or a PAI-1 polymorphism result does, one this review’s pre/post-PGT-A framework was not originally built to capture, since that framework assumes a stable, one-time-determinable factor rather than a state that may itself need repeat measurement to trust [62,63].

This is precisely the confound the embryo-aware framework from Section 3 was built to remove, and precisely the confound the receptivity literature has been slowest to remove from its own study designs. Until a study tests a defined receptivity gene panel, or an ERA-style classification, exclusively against outcomes in a cohort of confirmed euploid transfers, and does so with attention to whether that classification itself replicates on repeat testing, the true independent contribution of endometrial gene expression timing to genuine, embryo-excluded implantation failure remains mechanistically plausible and biologically well characterized at the level of individual genes like LIF and KLF12, but empirically unresolved at the population level in exactly the sense this review has tracked through every section before it. Figure 2 places the molecular mechanisms discussed across Section 4, Section 5, Section 6 and Section 7 onto the sequential biological steps of implantation, from gametogenesis through spiral artery remodeling, showing at which specific step each proposed genetic factor is thought to act.

## 8. Molecular Biomarkers and Translational Potential

Every mechanism covered so far raises the same practical question. Can any of it actually be measured, before a transfer, in a way that changes what a clinician does next? A biomarker is not just an association from a case-control study. It has to predict something in an individual patient, reliably enough, and consistently enough across repeat measurements, to justify acting on it. This section takes every genetic and molecular finding discussed in Section 4, Section 5, Section 6 and Section 7 and asks the same question of each: how far has it actually traveled from association to usable clinical tool.

### 8.1. Which Candidates Have Cleared the First Bar

Two categories of biomarkers discussed in this review have reached at least a meta-analytic level of evidence: the PAI-1 4G/4G genotype (Section 5.2) and the KIR AA haplotype (Section 6.2), both replicated across independent cohorts rather than resting on a single study [12,15]. Both are genotyping assays run from a single blood draw, using PCR-based methods already established in clinical genetics laboratories, requiring no specialized equipment and no repeat sampling across a treatment cycle, since a germline genotype does not change from one cycle to the next.

This stability is itself a potential translational advantage over the endometrial receptivity biomarkers discussed in Section 7, though this remains to be confirmed rather than established. A PAI-1 genotype result from one blood draw, taken at any point before treatment begins, remains valid for the rest of a patient’s reproductive life; an ERA classification from a single endometrial biopsy may not carry the same durability, as the test–retest discrepancy discussed in Section 7.4 illustrates [63]. If confirmed at cohort scale, a biomarker needing re-verification before every treatment cycle, and capable of giving a different answer on retesting even when the underlying condition has not changed, would carry a fundamentally different cost, logistics, and interpretive burden than one measured once and trusted thereafter.

The HLA-G and ERAP1-HLA-C-KIR findings from Section 6 sit in an intermediate position between these two extremes. The combined ERAP1-HLA-C model discussed in Section 6.3 is, mechanistically, the richest biomarker model in this review, capturing a genuine three-locus interaction, maternal enzyme genotype, embryonic HLA-C genotype, and by extension maternal KIR genotype, rather than a single-locus main effect [16]. But a three-gene interaction model is considerably harder to translate into a clinical test than a single genotype call: it requires typing maternal ERAP1 status, typing embryonic or paternal HLA-C status, and cross-referencing both against a lookup table of protective and non-protective combinations, none of which has yet been independently replicated in a second population.

### 8.2. Where Biological Plausibility Outpaces Clinical Validation

The KLF12-LIF axis discussed in Section 7.2 is the clearest example in this review of a mechanism with strong molecular characterization and essentially no biomarker translation. Huang and colleagues demonstrated the transcriptional repression mechanism directly in human endometrial tissue from RIF patients, with a clear, previously validated molecular logic connecting elevated KLF12 to suppressed LIF [17]. But none of this has been converted into a standardized clinical assay: KLF12 quantification in an endometrial biopsy has not been tested prospectively as a predictor of implantation outcome, and no clinical cutoff separating a normal from an abnormal result exists. The gap here is not biological plausibility, which is high; it is the absence of assay development, threshold-setting, and prospective validation that separates a mechanistic finding from an orderable biomarker.

The ANXA5 M2 haplotype discussed in Section 5.2 illustrates a related but distinct translational problem: not absent validation, but inconsistent validation across populations, with the German cohort finding not replicated in independent Northern European studies [13]. A biomarker that predicts outcome in one population and not in a closely related one is not simply a weaker version of a working biomarker. It signals that predictive value cannot currently be assumed to generalize, and that clinical use would need separate validation in each population where it might be deployed, an added burden the more consistently replicated PAI-1 and KIR findings do not carry to the same degree.

### 8.3. The Structural Barrier Common to All of Them

This shared limitation has a direct practical consequence. A clinician cannot currently tell a patient with a PAI-1 4G/4G genotype, a KIR AA haplotype, or an elevated KLF12 signature that her risk of implantation failure specifically after a confirmed euploid transfer is elevated by any evidence-based amount, because that comparison has not been made in the published literature. What can be said is that these markers are associated with RIF as defined under the older, embryo-blind framework, a population that likely contains a substantial admixture of patients whose true problem was an unrecognized aneuploid embryo.

This matters directly for how a positive result should be counseled to a patient in clinical practice. Offering a PAI-1 or KIR test to a woman with RIF, and interpreting a positive result as if it applied cleanly to her situation, assumes an effect size measured in a mixed population applies unchanged to the narrower population an embryo-aware definition would isolate. That assumption has never been tested. The true effect size in a ploidy-confirmed population could be larger, if the mechanism is genuinely independent of the embryo and the mixed population simply diluted a real signal with aneuploidy-driven noise. It could equally be smaller, or absent altogether, if a meaningful fraction of the “RIF” cases originally used to establish the association were in fact aneuploidy-driven failures unrelated to the biomarker being tested. Both directions of bias are mechanistically plausible given what Section 4, Section 5, Section 6 and Section 7 have shown, and the existing literature offers no way to determine which direction, if either, actually applies to any specific one of these biomarkers.

### 8.4. What a Translationally Complete Biomarker Study Would Require

Closing this gap for any single biomarker discussed in this review would require a specific study design that, as far as the literature search underlying this review could determine, has not yet been published for any of them. The study would need to enroll a cohort in which every transferred embryo carries a documented, PGT-A-confirmed euploid result, removing embryonic aneuploidy as a competing explanation for implantation failure at the outset rather than after the fact. It would need to measure the candidate biomarker, whether a fixed germline genotype like PAI-1 or KIR, or a tissue expression signature like KLF12 or an ERA-style panel, prospectively, before the transfer whose outcome is being predicted. And for any biomarker whose reliability across repeated measurements is itself in question, as the Ota discrepancy case raises for ERA-based classification, the study would need to include repeat testing within the same patient to establish whether the biomarker result is a stable trait or a fluctuating state before it is used to guide a clinical decision.

No study meeting all three of these conditions currently exists for any of the biomarkers discussed across Section 4, Section 5, Section 6, Section 7 and Section 8 of this review. Until one does, the translational status of this entire field can be summarized honestly as follows: several individual mechanisms are well characterized at the molecular level; one or two associations, PAI-1 and KIR haplotype in particular, have reached a reasonable threshold of statistical replication across independent cohorts, but none has been tested in the specific population, PGT-A-confirmed euploid transfers, that would establish whether it predicts genuine non-embryonic implantation failure rather than simply correlating with a mixed population in which unrecognized embryonic aneuploidy was doing an unknown share of the work.

## 9. Current Controversies and Knowledge Gaps

This section pulls together, deliberately, without new evidence, everything Section 4, Section 5, Section 6, Section 7 and Section 8 established one mechanism at a time. The point is not to repeat those findings. It is to look at them side by side and ask what pattern emerges when the four domains are compared directly against each other, rather than read in isolation.

### 9.1. The Same Gap, Four Times, for Four Different Reasons

Every domain covered in this review shares the identical structural gap: no study has tested its central claim in a cohort restricted to confirmed euploid embryo transfers. But this gap does not have the same cause, or the same consequence, in each domain, and treating it as a single uniform problem would obscure more than it reveals.

For parental karyotype, discussed in Section 4, the gap exists because PGT-A does not remove the phenomenon from view when a structural rearrangement is present; it instead reclassifies the case as embryonic aneuploidy, migrating it out of the RIF category rather than testing it within it (Section 4.2). This is not a gap future study design alone can close. It requires a fundamentally different cohort, built around parental karyotype status and PGT-SR outcomes specifically, rather than around RIF as a downstream label.

For thrombophilia, discussed in Section 5, the gap exists for a more ordinary reason: nobody has stratified existing case–control cohorts by embryo ploidy, even though doing so is technically straightforward in any center that already offers PGT-A. This is the most fixable of the four gaps. It requires no new mechanism, no new assay, and no new patient population, only a retrospective or prospective re-analysis of existing thrombophilia data cross-referenced against ploidy status already sitting in clinic records.

For the immunogenetic mechanisms discussed in Section 6, the gap is complicated by the fact that the relevant genotype, HLA-C in particular, belongs partly to the embryo rather than purely to the mother. An aneuploid embryo could carry an entirely normal HLA-C genotype, meaning ploidy and immunogenetic risk are not obviously correlated, but this independence has never actually been demonstrated, only assumed by default in the absence of any study checking it directly.

Retrospective cohorts restricting analysis to PGT-A-confirmed euploid transfers now exist for this domain specifically, Cozzolino and colleagues in a multicenter series of 2598 patients and Neves and colleagues in a smaller single-center comparison, but both found no consistent benefit from ERA-guided timing over standard transfer, leaving the central question open rather than answered [67,68]. For endometrial receptivity, the gap is compounded by the reliability problem discussed in Section 7.4: a biomarker that gives different answers on retesting cannot be validated against outcome with any confidence, regardless of how carefully the embryo side of the equation is controlled.

### 9.2. Where the Mechanisms Might Interact Rather than Simply Coexist

Section 4, Section 5, Section 6 and Section 7 were written as four separate domains, and for the most part the existing literature treats them that way too, testing one mechanism per study without reference to the others. But nothing in the underlying biology requires these mechanisms to act independently, and at least two plausible interactions surfaced across the preceding sections that no study currently addresses directly.

The first involves KIR genotype and embryo quality. Section 6.4 raised the possibility that a maternal KIR AA genotype might specifically affect patients who are also, for unrelated clinical reasons, more likely to be offered marginal or mosaic embryos, a confound existing case–control designs cannot resolve because they do not measure embryo quality with sufficient granularity to test for it. If this interaction is real, then part of the KIR AA association reported by Maftei and colleagues could reflect a shared upstream cause, rather than KIR genotype acting as an independent risk factor in its own right.

The second involves thrombophilic genotype and embryo viability at the margin. Section 5.4 raised the mirror-image possibility: that a hypofibrinolytic maternal genotype like PAI-1 4G/4G might specifically increase the implantation window’s tolerance for a marginally competent embryo that a more restrictive hemostatic environment would have prevented from implanting at all. If true, this would mean the PAI-1 association, rather than being diluted by an unstratified cohort as Section 5.4 initially framed it, could actually be inflated by exactly the embryos a euploidy-restricted cohort would remove, producing the opposite direction of bias from what a naive dilution model would predict.

Neither interaction has been tested. Both are mechanistically plausible given what is already known about the individual pathways. Their existence, even as untested possibilities, means the pre/post-PGT-A stratification this review has argued for throughout is necessary but may not be sufficient on its own. A study that simply restricts its cohort to euploid transfers, without also considering how a genetic variant might interact with the embryo selection process that produced that euploid classification in the first place, could still misattribute an interaction effect to a main effect, or vice versa.

### 9.3. A Field-Wide Denominator Problem

Underlying all four domains is a problem that predates PGT-A entirely and has not been resolved by its introduction: the definitional instability of RIF itself, discussed at length in Section 3. Every prevalence figure, every odds ratio, every association reported across Section 4, Section 5, Section 6, Section 7 and Section 8 was generated using a study-specific definition of RIF, drawn from a landscape in which, as documented by the survey work underlying the Lugano consensus, clinicians and embryologists have never agreed on a single operational definition. This means the denominator against which every numerator in this review was calculated is itself inconsistent across studies, independent of the ploidy question this review has focused on. Two studies reporting different odds ratios for the same PAI-1 genotype may be measuring genuinely different populations, not because one used PGT-A and the other did not, but because their RIF inclusion criteria selected for different underlying severity or duration of infertility altogether.

This compounds, rather than substitutes for, the ploidy problem this review has tracked throughout. Fixing the denominator alone, by adopting a single consistent RIF definition across future studies, would not resolve the embryo-blind versus embryo-aware distinction established in Section 3. And fixing the ploidy problem alone, by restricting future cohorts to confirmed euploid transfers, would not resolve the definitional inconsistency documented by the clinician survey work discussed earlier. Both problems need to be solved together, in the same study design, for any of the associations reviewed in Section 4, Section 5, Section 6, Section 7 and Section 8 to be tested on genuinely comparable footing across future replications. Table 2 lists the primary studies discussed across Section 4, Section 5, Section 6, Section 7 and Section 8, indicating for each whether embryonic aneuploidy was excluded at study entry, the point on which the argument of this review has consistently turned.

## 10. Discussion

This review set out to answer one question: how much of the genetic architecture proposed for recurrent implantation failure survives once embryonic aneuploidy is removed as the default explanation for a failed transfer. Having gone through four separate domains individually, this section brings them together and asks that question directly, study by study, rather than mechanism by mechanism.

### 10.1. What the Definitional Literature Actually Establishes

Coughlan et al. (2014) and the ESHRE PGD Consortium (2005) built the field’s original operational definitions of RIF around embryo count and morphological grade alone, with no mechanism to check chromosomal status [1,2]. Ata et al. (2021), Pirtea et al. (2023) for the Lugano consensus, Garneau and Young (2021), and Gill et al. (2024) each challenged this framework from a different angle statistically, definitionally, and empirically converging on the same interpretation discussed in Section 3: that a meaningful share of what earlier decades called RIF may have been embryonic rather than maternal in origin, though the exact proportion cannot be determined from currently available data [3,4,5,6]. This has a direct consequence for how every association reviewed in Section 4, Section 5, Section 6, Section 7 and Section 8 should be weighted: none was generated in the population these five sources have shown to be the biologically meaningful one. They were generated in the older, embryo-blind population that may include a substantial, though unquantified, share of unrecognized embryonic aneuploidy. This does not make the associations false; it makes their generalizability to genuine, ploidy-confirmed implantation failure an open question rather than a settled one.

### 10.2. Parental Karyotype: A Mechanism That Does Not Disappear, It Migrates

Upadhyay et al. (2024) demonstrated, in a single but mechanistically clear case, that a complex chromosome rearrangement can explain a decade of unexplained failure once high-resolution karyotyping and targeted PGT-SR were applied [39]. The four prevalence studies discussed in Section 4.3, De Sutter et al. (2012), Mozdarani et al. (2008), Petrovic et al. (2024), and Asgari et al. (2025), together establish that structural rearrangements are neither rare nor uniformly defined across cohorts, but all relied on conventional G-banding rather than higher-resolution methods, meaning every reported prevalence figure likely underestimates the true rate to a comparable degree [8,9,10,11].

The more consequential problem, though, is not the prevalence spread. It is that De Sutter et al. (2012), Mozdarani et al. (2008), Petrovic et al. (2024), and Asgari et al. (2025) all applied the embryo-blind RIF definition criticized by Ata et al. (2021) and Pirtea et al. (2023) [3,4,8,9,10,11]. A translocation carrier identified in any of these four cohorts, transferring predominantly unbalanced-segregation embryos, would today generate PGT-A results consistent with recurrent embryonic aneuploidy, which, under an embryo-aware diagnostic framework, would place these cases outside RIF in the narrower sense this review has tried to isolate. This means the entire parental karyotype literature reviewed here answers a question adjacent to, but not identical with, the one this review is asking. Upadhyay et al. (2024) show the mechanism is real and clinically actionable [39]. De Sutter et al. (2012) and the three studies that followed show it is not rare [8]. None of the five shows whether it contributes to implantation failure independent of the aneuploidy it produces, because the two outcomes, aneuploid embryo and failed implantation, cannot be separated once a rearrangement is already generating the aneuploidy directly.

### 10.3. Thrombophilia: A Real Association Without a Demonstrated Developmental Stage

Zeng et al. (2021) reported the single strongest quantitative association in this review for the PAI-1 4G/4G genotype (Section 5.2), and Rogenhofer et al. (2021) reported a comparable, though less extensively replicated, elevated risk for the ANXA5 M2 haplotype [12,13]. The ESHRE Working Group (2023), reviewing this same evidence, nonetheless concluded that the role of thrombophilia in RIF remains increasingly challenged, a conclusion that appears, at first reading, to sit in tension with the pooled odds ratio Zeng et al. (2021) reported [12,30].

That tension resolves once the developmental-stage question raised in Section 5.3 is taken seriously. Zeng et al. (2021) established a statistical association between genotype and a clinical outcome labeled RIF [12]. It did not establish, and could not establish from case–control data alone, whether the proposed mechanism, impaired trophoblast invasion through hypofibrinolysis, acts early enough to block initial attachment, or whether it acts later, contributing instead to the placental insufficiency phenotypes it is more directly implicated in elsewhere in the literature, such as preeclampsia. The ESHRE Working Group’s (2023) caution, read this way, is not a rejection of Zeng et al. (2021) findings [12,30]. It is a recognition that a real genetic association with an outcome labeled RIF does not automatically confirm the specific implantation-stage mechanism the association is usually invoked to support. Rogenhofer et al. (2021) equal-risk paternal finding adds a further complication neither study resolves: if paternal ANXA5 genotype matters as much as maternal genotype, the mechanism may operate partly through the embryo’s own trophoblast tissue rather than purely through the maternal decidual environment, a possibility that would place ANXA5 closer to the immunogenetic category discussed in Section 6 than to a purely maternal thrombophilic mechanism [13].

### 10.4. Immunogenetics: The Strongest Mechanistic Coherence, Still Untested Against Ploidy

Hu et al. (2022) reported a paternal HLA-G allele independently associated with RIF risk, implicating the embryo’s own paternally inherited HLA-G expression rather than maternal genotype alone [14]. Maftei et al. (2023) demonstrated a clean dose–response pattern across KIR haplotypes that the receptor biology discussed in Section 6.2 predicts mechanistically rather than merely describes statistically [15,55]. Piekarska et al. (2022) extended this further with the three-locus ERAP1-HLA-C interaction discussed in Section 6.3, which neither Hu et al. (2022) nor Maftei et al. (2023) tested in isolation [14,15,16].

Of all four domains reviewed in this paper, the immunogenetic literature represented by Hu et al. (2022), Maftei et al. (2023), and Piekarska et al. (2022) offers the most internally consistent mechanistic story, because each additional layer of complexity, from single HLA-G alleles to KIR haplotype to the three-way ERAP1-HLA-C interaction, extends rather than contradicts the layer before it [14,15,16]. But Piekarska et al. (2022), Maftei et al. (2023), and Hu et al. (2022) share the same limitation as every other domain in this review: none restricted its cohort to confirmed euploid transfers [14,15,16]. This is a more serious gap here than for thrombophilia, because Hu et al. (2022) and Piekarska et al. (2022) both study genotypes that are partly embryonic, meaning an aneuploid embryo in either study’s cohort could, in principle, carry a normal or abnormal HLA-C or HLA-G genotype independent of its ploidy status, an independence Section 6.4 flagged as assumed rather than demonstrated [14,16].

### 10.5. Endometrial Receptivity: Mechanistically Rich, Structurally the Least Resolved

Huang et al. (2018) provided the clearest single-gene mechanistic demonstration in this review (Section 7.2), and Opuchlik et al. (2025) found this pattern consistent with a 68-patient RIF cohort predominantly classified as pre-receptive or early-receptive on standard biopsy timing [17,19]. Amin et al. (2022) came closest among all studies reviewed here to the ideal design this review has argued for, combining ERA-guided transfer timing with PGT-A screening in a subset of a 291-patient cohort [18].

Even so, Amin et al. (2022) applied PGT-A only to a subset of the full cohort, leaving the majority of the sample subject to the same embryo-ploidy ambiguity affecting every other study in this review [18]. The reliability question raised by the Ota discrepancy case (Section 7.4) means that even a hypothetical future study restricting its cohort to confirmed euploid transfers would still need to resolve whether that discrepancy reflects a general assay limitation or an isolated case, before any ploidy-stratified receptivity finding could be trusted at face value [63].

### 10.6. Reading the Four Domains Against Each Other

Placed side by side, these four bodies of evidence do not support a uniform conclusion about the genetics of RIF. Upadhyay et al. (2024), De Sutter et al. (2012), and the karyotype literature that follows them describe a mechanism that is real, well characterized, and clinically actionable through PGT-SR, but one that an embryo-aware diagnostic framework in the PGT-A era has arguably moved out of the RIF category entirely rather than tested within it [8,39]. Zeng et al. (2021) and Rogenhofer et al. (2021) describe an association strong enough to survive meta-analytic pooling, yet the ESHRE Working Group (2023) correctly notes that statistical association has outrun mechanistic confirmation at the specific developmental stage implantation requires [12,13,30]. Hu et al. (2022), Maftei et al. (2023), and Piekarska et al. (2022) describe the most mechanistically coherent multi-locus system in this review, built on receptor biology that predicts its own findings before the data confirm them, yet remain untested against ploidy status as in every other domain [14,15,16]. Huang et al. (2018), Opuchlik et al. (2025), and Amin et al. (2022) describe a mechanism operating on a maternal tissue that changes state cycle to cycle, a property none of the other three domains share, and one that Ota et al. (2022) suggests may complicate biomarker validation even after the ploidy question is eventually addressed [17,18,19,63].

The honest summary, then, is not that RIF genetics is an empty field. It is that four mechanistically distinct pathways, each grounded in plausible molecular biology and each supported by statistical association in the historical, embryo-blind literature, have each been tested in a population that Ata et al. (2021) and Gill et al. (2024) have shown is not the population these pathways are actually meant to explain [3,6]. None of the four has yet been shown to contribute independently to implantation failure in a cohort where embryo euploidy was confirmed at enrollment. Correcting this will not require abandoning any of the findings reviewed in Section 4, Section 5, Section 6, Section 7 and Section 8. It will require retesting each of them, individually and possibly in combination given the interactions raised in Section 9.2, in the narrower cohort that a PGT-A-confirmed definition of RIF, as argued by Ata et al. (2021) and empirically supported by Gill et al. (2024), has finally made possible to study [3,6].

## 11. Future Research Directions

Every gap identified across Section 4, Section 5, Section 6, Section 7, Section 8, Section 9 and Section 10 points toward the same missing study design. This section makes that design explicit, mechanism by mechanism, rather than leaving it as an abstract recommendation.

### 11.1. A Prospective, Ploidy-Stratified RIF Cohort as the Foundational Study

The single most consequential study this field could run does not yet exist in any form. It would enroll women meeting a modern, statistically grounded RIF definition, following the framework established by Ata et al. (2021) rather than the older embryo-count criteria used by Coughlan et al. (2014), and it would require, as an entry criterion rather than a subgroup analysis performed afterward, that every transferred embryo in the patient’s history carry a documented PGT-A-confirmed euploid result [1,3]. This single design choice would resolve the structural limitation shared by every study reviewed in Section 4, Section 5, Section 6, Section 7 and Section 8, since none of them, including Zeng et al. (2021) meta-analysis of eight thrombophilia cohorts and Maftei et al. (2023) prospective KIR haplotype study, applied this restriction at enrollment [12,15].

Such a cohort would also need to be large enough to detect the effect sizes reported in the existing embryo-blind literature, should they persist in a ploidy-confirmed population. Maftei et al. (2023) reported an adjusted odds ratio of 4.15 for the KIR AA haplotype in a cohort large enough to reach statistical significance despite being a single-center study; a future ploidy-stratified replication would need comparable or greater power, since restricting enrollment to confirmed euploid transfers necessarily shrinks the eligible population relative to an embryo-blind design, and a smaller true effect size in the euploid-restricted population, as discussed in Section 8.3, cannot be ruled out in advance [15].

### 11.2. Testing the Interactions Raised in Section 9.2, Not Just the Main Effects

A ploidy-stratified cohort that tests each mechanism in isolation would still miss the interaction effects raised in Section 9.2. Future work needs to test, directly, whether the KIR-embryo-quality interaction proposed in Section 9.2 is real, by measuring embryo morphological and chromosomal parameters with enough granularity to distinguish a euploid-but-marginal embryo from a robustly euploid one, something Maftei et al. (2023) design did not attempt. Likewise, the possibility raised in Section 9.2 that PAI-1 4G/4G genotype might increase tolerance for marginal embryos, rather than acting independently of embryo quality as Zeng et al. (2021) implicitly assumed, requires a study design that deliberately varies or measures embryo quality within a ploidy-confirmed cohort, rather than treating euploidy as a single binary category once confirmed [12,15].

The three-locus interaction reported by Piekarska et al. (2022), combining maternal ERAP1 genotype with paternal or embryonic HLA-C genotype, represents the most complex model discussed in this review and the one furthest from independent replication [16]. A future study would need, at minimum, to test this same combination in a second, independent population before treating the reported odds ratios of 0.343 and 0.442 as generalizable, and would ideally extend the model to include maternal KIR genotype directly, since Piekarska et al. (2022) study measured ERAP and HLA-C but did not simultaneously genotype KIR haplotype in the same patients that Maftei et al. (2023) study characterized separately [15,16].

### 11.3. Resolving the Reliability Question Before the Ploidy Question, for Endometrial Receptivity Specifically

Endometrial receptivity biomarkers carry an additional prerequisite that the other three domains do not. Before any ploidy-stratified study of ERA classification or of the KLF12-LIF mechanism described by Huang et al. (2018) can be meaningfully designed, the reliability question raised by the single case reported by Ota et al. (2022) needs a proper answer at cohort scale rather than a single documented instance [17,63]. This would require a study that performs repeat receptivity testing, whether by ERA, by the 67-gene beREADY panel used by Opuchlik et al. (2025), or by direct KLF12 quantification, on the same patients across multiple cycles, before any single measurement from any of these platforms is used to guide a clinical transfer decision in a future ploidy-stratified trial [19]. Without this step first, a future study combining PGT-A confirmation with receptivity testing would risk repeating the same interpretive ambiguity Amin et al. (2022) study left partially unresolved, since a single receptivity measurement of uncertain reliability cannot be trusted as a stable predictor variable regardless of how well embryo ploidy is controlled elsewhere in the design [18].

### 11.4. A Combined Denominator: Solving Definition and Ploidy Together

Section 9.3 argued that the definitional inconsistency documented across the clinician survey work underlying the Lugano consensus and the embryo-ploidy problem this review has tracked throughout are separate problems requiring simultaneous correction. Future studies in this field should adopt a single, explicit, published RIF definition, ideally the probabilistic framework proposed by Ata et al. (2021) or the more clinically pragmatic 60% threshold proposed by the ESHRE Working Group (2023), and should report, transparently, which threshold was used, so that future systematic reviews can compare studies against a known and stated denominator rather than the mixture of criteria that has made direct comparison across the studies discussed in Section 4, Section 5, Section 6, Section 7, Section 8, Section 9 and Section 10 difficult throughout this review [3,30].

Only a study meeting all of these conditions together, a stated modern RIF definition, mandatory PGT-A confirmation of euploidy at enrollment, adequate power to detect effects smaller than those reported in embryo-blind cohorts, explicit testing of gene-embryo interactions rather than main effects alone, and, for receptivity biomarkers specifically, prior establishment of test–retest reliability, would finally answer the question this review set out to ask. Until such a study exists, the honest position, for each of the four mechanisms reviewed here, is the one reached independently in Section 4, Section 5, Section 6 and Section 7: molecularly plausible, statistically associated with RIF as historically defined, and empirically untested in the population that PGT-A has made possible to study for the first time.

## 12. Conclusions

This review asked whether the genetic evidence behind recurrent implantation failure still holds now that PGT-A can rule out embryonic aneuploidy. Across all four domains examined, the answer is consistent: the mechanisms are real, but the evidence has not caught up to what PGT-A now makes possible to test.

Parental structural rearrangements are the most mechanistically clear. But PGT-A results for these cases are, by definition, aneuploid; under an embryo-aware diagnostic framework, this places the mechanism in the recurrent embryonic aneuploidy category rather than RIF, moving it outside the category this review set out to study.

Thrombophilic variants carry the strongest statistical signal, particularly PAI-1. But statistical association has outrun mechanistic confirmation of when, in the implantation process, this pathway actually acts.

Immunogenetic findings, especially the KIR-HLA-C-ERAP interaction, offer the richest internal biological logic of any domain reviewed. Even so, this system has never been tested against confirmed embryo ploidy.

Endometrial receptivity remains the least resolved. Its biology is well described at the gene level, and a single case report raises the hypothesis-generating possibility that even repeated measurements in the same patient may not be reproducible, a question that has not yet been tested at cohort scale.

The unifying gap is simple to state. No study in any of these four areas has tested its central claim in a population where embryo euploidy was confirmed at enrollment. Until that study exists, for each mechanism, the genetics of RIF remains plausible, associated, and unproven in the one population that now matters most. A residual layer of uncertainty attaches to this evidence audit as well: as noted in Section 2, embryo-ploidy status was not always reported explicitly, and some studies were assigned to the pre-PGT-A or PGT-A-confirmed category by inference from their methods rather than by the original authors’ own statement, a limitation worth weighing alongside the conclusions above.

## Figures and Tables

**Figure 1:**
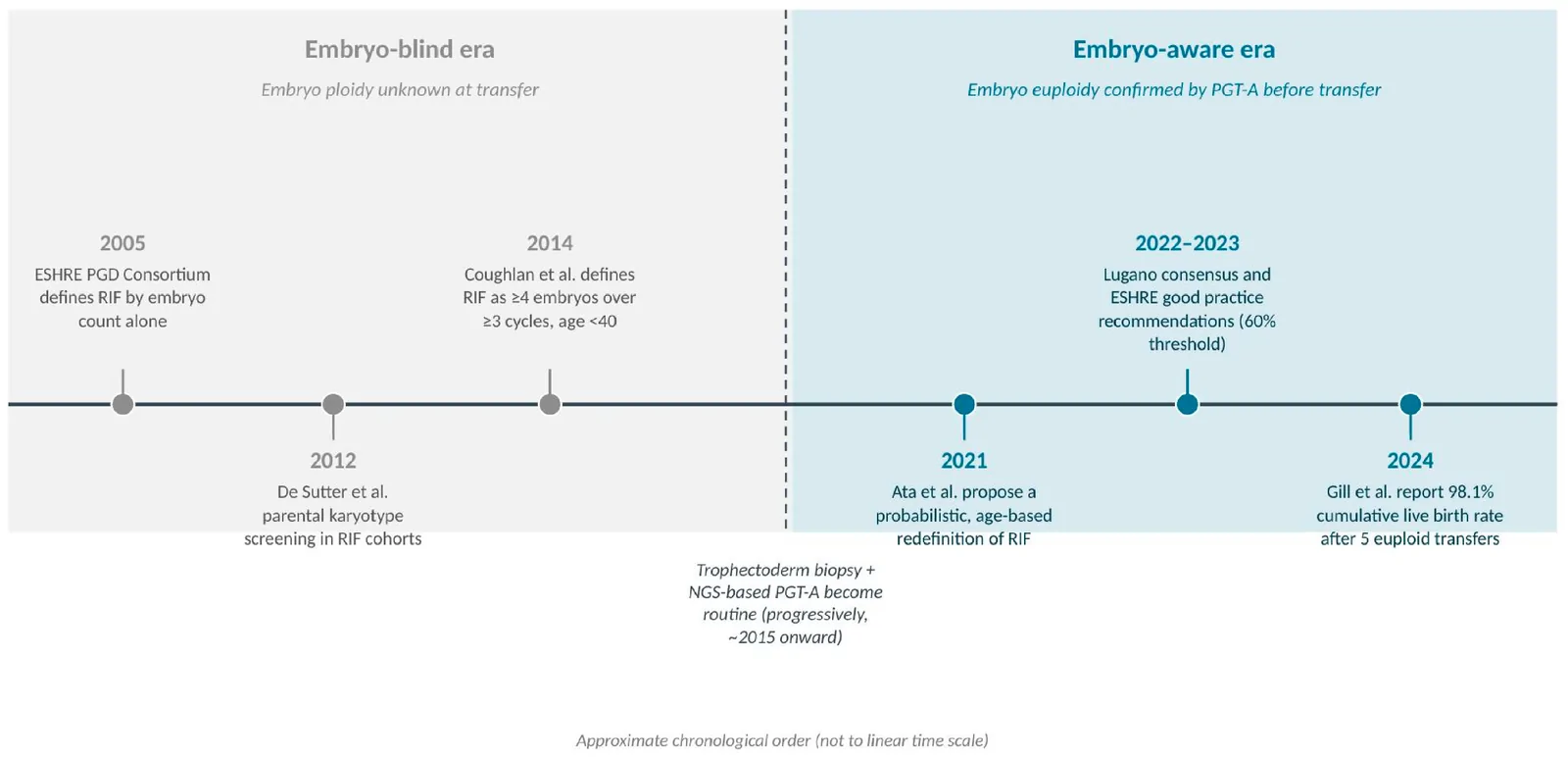
Timeline of the evolution of definitions of recurrent implantation failure (RIF) and the clinical introduction of preimplantation genetic testing for aneuploidy (PGT-A). Figure 1. Chronological summary of key milestones in the definition of RIF, positioned relative to the clinical adoption of trophectoderm biopsy combined with next-generation sequencing (NGS)-based PGT-A (2013–2015), shown as the dashed vertical divider. Milestones to the left of the divider (grey) were established under an embryo-blind framework, in which embryo ploidy was not assessed at the time of transfer. Milestones to the right (teal) reflect the embryo-aware framework enabled by routine PGT-A, in which embryo euploidy is confirmed before transfer. Spacing along the timeline reflects approximate chronological order and is not drawn to a linear time scale [1,2,3,6,8,30].

**Figure 2:**
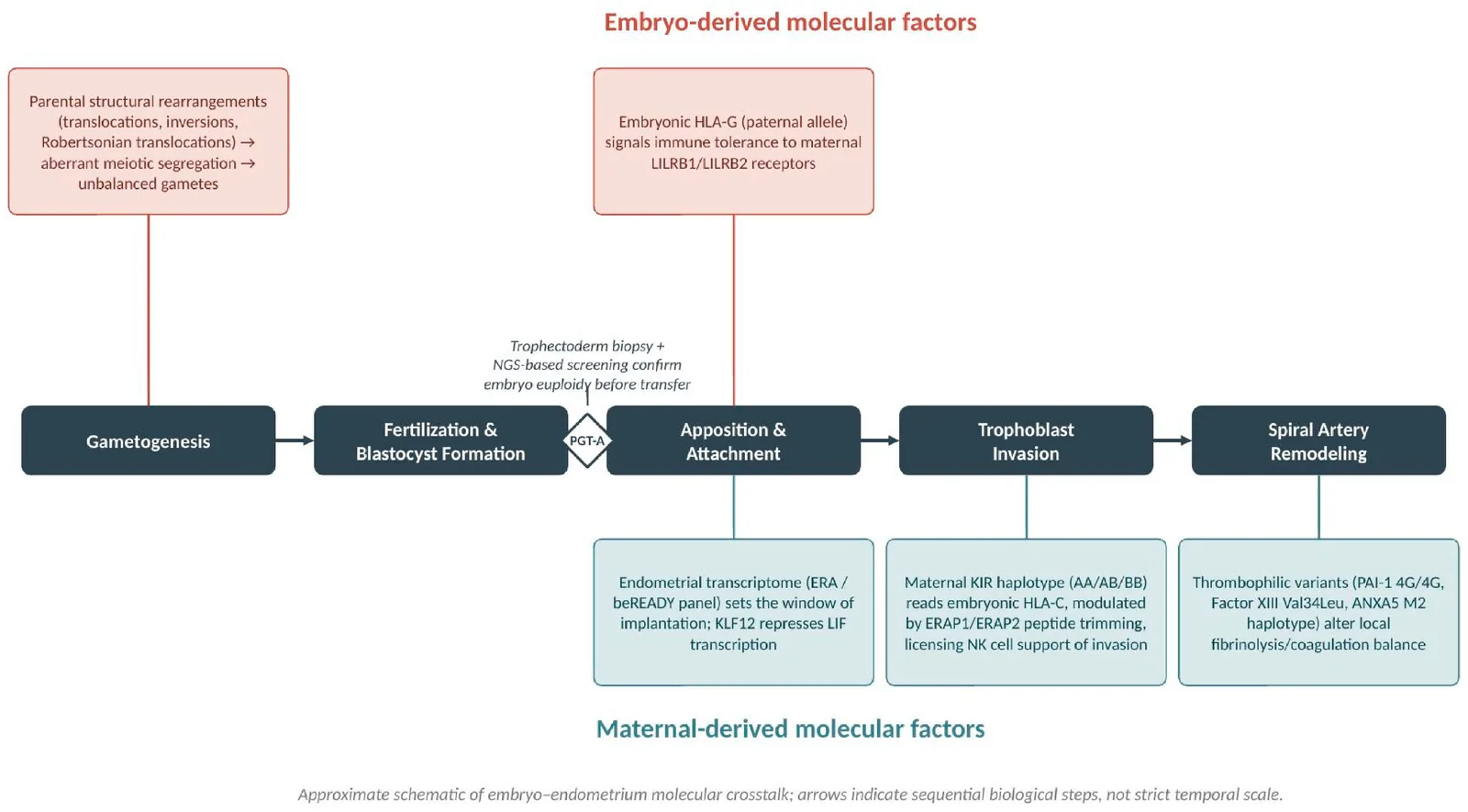
Molecular crosstalk between the embryo and the endometrium across sequential steps of implantation, annotated with the genetic and molecular mechanisms discussed in this review. Figure 2. Schematic representation of the sequential biological steps of human implantation, from gametogenesis through spiral artery remodeling. Embryo-derived molecular factors (top, terracotta) are annotated above the corresponding step at which they act: parental structural chromosomal rearrangements disrupt meiotic segregation during gametogenesis. PGT-A confirms embryo euploidy at the blastocyst stage before transfer, and embryonic HLA-G, inherited in part from the paternal genome, signals immune tolerance during attachment. Maternal-derived molecular factors (bottom, slate blue) are annotated below the corresponding step: endometrial transcriptomic receptivity and the KLF12-LIF axis govern the timing of attachment; maternal KIR haplotype, modulated by ERAP1/ERAP2 peptide trimming, reads embryonic HLA-C to license natural killer cell support of trophoblast invasion; and thrombophilic gene variants alter the local fibrinolysis-coagulation balance during spiral artery remodeling. Arrows indicate the sequence of biological steps and are not drawn to a strict temporal scale.

**Table 1:** Genetic and molecular mechanisms proposed as contributors to recurrent implantation failure, their reported clinical associations, and the current availability of PGT-A-confirmed evidence.

Category	Gene/Factor	Proposed Molecular Mechanism	Reported Association (Study, Effect Size)	PGT-A-Confirmed Evidence Available
Parental chromosomal rearrangement, [8,9,10,11]	Balanced reciprocal translocations, Robertsonian translocations, inversions	Aberrant meiotic segregation (adjacent-1, adjacent-2, 3:1) of quadrivalent or trivalent configurations, producing structurally unbalanced gametes	Prevalence 2.1–9.5% across cohorts	No
Thrombophilia, [12]	*SERPINE1* (PAI-1) 4G/5G	4G allele reduces repressor binding at promoter, increasing PAI-1 transcription and inhibiting plasmin-mediated trophoblast invasion	OR 2.79 (95% CI 1.53–5.08) for 4G/4G genotype	No
Thrombophilia, [13]	*ANXA5* M2 haplotype	Reduced annexin A5 promoter activity, decreasing trophoblast surface shielding from procoagulant phospholipids	Increased relative risk, equal maternal/paternal carriage effect	No
Immunogenetics, [14]	*HLA-G* (14 bp ins/del, −725 promoter variant)	Altered mRNA stability and transcription affecting soluble HLA-G–mediated inhibitory signaling to maternal LILRB1/LILRB2	Maternal HLA-G*010101 and paternal HLA-G*010102 alleles associated with RIF risk	No
Immunogenetics, [15]	*KIR* AA/AB/BB haplotype	Haplotype-dependent balance of inhibitory versus activating signaling in uterine NK cells via HLA-C recognition	AA: aOR 4.15 (95% CI 1.39–6.50) for miscarriage; BB: 87.5% live birth rate	No
Immunogenetics, [16]	*ERAP1* rs27044 combined with *HLA-C* group	ERAP1-dependent peptide trimming alters HLA-C peptide cargo, modulating KIR engagement	OR 0.343–0.442 for protective genotype combinations	No
Endometrial receptivity, [17]	*KLF12*/*LIF* axis	KLF12 transcriptional repression of LIF promoter, impairing trophectoderm–epithelial attachment signaling	Elevated KLF12, decreased LIF in RIF endometrium versus controls	No
Endometrial receptivity, [18,19]	ERA/beREADY transcriptomic panel (67–238 genes)	Displacement of the transcriptomic window of implantation relative to standard transfer timing	70/68 RIF patients classified as pre-/early-receptive rather than receptive	Partial (subset only; retrospective PGT-A-confirmed subgroups)

Notes: OR, odds ratio; aOR, adjusted odds ratio; CI, confidence interval; PAI-1, plasminogen activator inhibitor-1; ANXA5, annexin A5; HLA, human leukocyte antigen; LILRB, leukocyte immunoglobulin-like receptor B; KIR, killer immunoglobulin-like receptor; ERAP, endoplasmic reticulum aminopeptidase; KLF12, Krüppel-like factor 12; LIF, leukemia inhibitory factor; RIF, recurrent implantation failure; ERA, endometrial receptivity array. “PGT-A-Confirmed Evidence Available” indicates whether the cited association has been tested in a cohort restricted to embryos with documented euploid status by preimplantation genetic testing for aneuploidy at the time of transfer.

**Table 2:** Summary of primary studies discussed in this review, by mechanistic category, indicating whether embryonic aneuploidy was excluded at study entry.

Category	Study (Author, Year)	Study Design	Sample Size	Embryo Ploidy Confirmed at Enrollment	Definition of RIF Used
Parental karyotype, [8]	De Sutter et al., 2012	Retrospective cohort	615 patients	No	≥3 failed IVF/ICSI cycles prior to karyotyping
Parental karyotype, [9]	Mozdarani et al., 2008	Case–control (RSA + RIF combined)	221 individuals	No	≥3 IVF/ICSI failures (mixed with RSA)
Parental karyotype, [11]	Petrovic et al., 2024	Retrospective cohort	100 patients	No	Not explicitly ploidy-stratified
Parental karyotype, [10]	Asgari et al., 2025	Case–control	100 couples (RIF), 200 controls	No	≥3 consecutive IVF/ICSI-ET failures
Parental karyotype, [39]	Upadhyay et al., 2024	Case report	1 couple	Yes (via PGT-SR)	10 prior IVF failures
Thrombophilia, [12]	Zeng et al., 2021	Systematic review/meta-analysis	8 studies, 1273 women	No (not reported in pooled studies)	Variable across pooled studies
Thrombophilia, [13]	Rogenhofer et al., 2021	Case–control	63 couples, 90 fertile controls	No	≥4 failed transfers of ≥4 blastocysts or ≥8 cleavage-stage embryos
Immunogenetics, [14]	Hu et al., 2022	Systematic review/meta-analysis	Multiple pooled studies	No	Variable across pooled studies
Immunogenetics, [15]	Maftei et al., 2023	Prospective cohort	Patients with RPL/RIF, 2020–2022	No	Not ploidy-restricted
Immunogenetics, [16]	Piekarska et al., 2022	Case–control	491 couples, 322 fertile controls	No	Not ploidy-restricted
Endometrial receptivity, [17]	Huang et al., 2018	Case–control (tissue/mechanistic)	RIF patients vs. fertile controls	No	Not explicitly stated
Endometrial receptivity, [19]	Opuchlik et al., 2025	Case–control	68 RIF patients, 49 controls	No	≥3 failed transfers of ≥1 BB-grade blastocysts
Endometrial receptivity, [18]	Amin et al., 2022	Retrospective cohort	291 RIF patients	Partial (PGT-A in subset only)	Not explicitly stated
Endometrial receptivity, [63]	Ota et al., 2022	Case report	1 patient	No	Repeated implantation failure with chronic endometritis
Endometrial receptivity, [67]	Cozzolino et al., 2020	Retrospective multicenter cohort	2598 patients (2110 moderate RIF; 488 severe RIF)	Partial (PGT-A subgroup within each severity stratum)	Moderate vs. severe RIF, stratified by number of prior failed transfers (per study-specific criteria)
Endometrial receptivity, [68]	Neves et al., 2019	Retrospective single-center cohort (2012–2018)	24 ERA + euploid-ET and 32 ERA + donor-ET, vs. 119 and 158 respective controls	Yes (Euploid-ET arm; PGT-A-confirmed)	≥1 failed euploid embryo transfer (autologous) or ≥2 failed donor embryo transfers
RIF definition/framework [3]	Ata et al., 2021	Statistical modeling	Population-level simulation	N/A (methodological)	Proposed probabilistic redefinition
RIF definition/framework [6]	Gill et al., 2024	Retrospective cohort	123,987 patients	Yes (all transfers euploid by PGT-A)	5 consecutive euploid blastocyst transfers

Notes: RIF, recurrent implantation failure; RSA, recurrent spontaneous abortion; RPL, recurrent pregnancy loss; PGT-A, preimplantation genetic testing for aneuploidy; PGT-SR, preimplantation genetic testing for structural rearrangements; N/A, not applicable. “Embryo Ploidy Confirmed at Enrollment” indicates whether the study restricted its cohort, at the point of inclusion, to patients with documented euploid embryo transfers, thereby excluding embryonic aneuploidy as a competing explanation for the reported outcome is included as a methodological reference point rather than a mechanistic study, since it establishes the population baseline against which the ploidy-unconfirmed studies above should be interpreted [6].

## Data Availability

No new data were created or analyzed in this study. Data sharing is not applicable to this article.
